# (*E*)-4-[4-(Dimethyl­amino)styr­yl]-1-methyl­pyridinium 4-bromo­benzene­sulfonate^1^

**DOI:** 10.1107/S1600536808043547

**Published:** 2009-01-08

**Authors:** Suchada Chantrapromma, Patcharaporn Jansrisewangwong, Rusmeenee Musor, Hoong-Kun Fun

**Affiliations:** a Crystal Materials Research Unit, Department of Chemistry, Faculty of Science, Prince of Songkla University, Hat-Yai, Songkhla 90112, Thailand; b X-ray Crystallography Unit, School of Physics, Universiti Sains Malaysia, 11800 USM, Penang, Malaysia

## Abstract

In the title compound, C_16_H_19_N_2_^+^·C_6_H_4_BrO_3_S^−^, the cation is nearly planar,  with a dihedral angle of 3.19 (15)° between the pyridinium and the dimethylaminophenyl rings, and exists in the *trans* configuration. In the crystal packing, the cations and anions are linked into chains parallel to the *c* axis. These chains are stacked along the *b* axis. The crystal is stabilized by weak C—H⋯O and C—H⋯π inter­actions, and a π–π inter­action is also observed with a *Cg*⋯*Cg* distance of 3.5675 (19) Å.

## Related literature

For background to NLO materials research, see: Chia *et al.* (1995 ▶); Sato *et al.* (1999 ▶); Nogi *et al.* (2000 ▶); Otero *et al.* (2002 ▶); Dittrich *et al.* (2003 ▶). For related structures, see, for example: Adachi *et al.* (1999 ▶); Chantrapromma *et al.* (2006 ▶; 2008 ▶); Jagannathan *et al.* (2007 ▶); Ogawa *et al.* (2008 ▶); Rahman *et al.* (2003 ▶); Yang *et al.* (2007 ▶). For comparison bond lengths, see Allen *et al.* (1987 ▶).
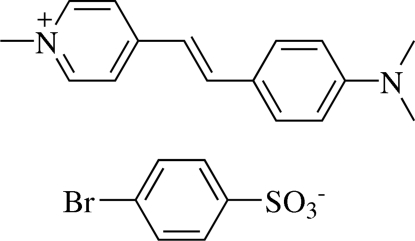


## Experimental

### 

#### Crystal data

C_16_H_19_N_2_^+^·C_6_H_4_BrO_3_S^−^
*M* *_r_* = 475.39
Monoclinic, 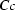

*a* = 10.3712 (4) Å
*b* = 10.9937 (5) Å
*c* = 17.9027 (8) Å
β = 92.442 (3)°
*V* = 2039.37 (15) Å^3^
*Z* = 4
Mo *K*α radiation
μ = 2.15 mm^−1^
*T* = 100.0 (1) K
0.49 × 0.31 × 0.11 mm

#### Data collection

Bruker SMART APEX2 CCD area-detector diffractometer
Absorption correction: multi-scan (*SADABS*; Bruker, 2005 ▶) *T*_min_ = 0.414, *T*_max_ = 0.787
13146 measured reflections
6479 independent reflections
4811 reflections with *I* > 2σ(*I*)
*R*_int_ = 0.043

#### Refinement

*R*[*F* ^2^ > 2σ(*F* ^2^)] = 0.046
*wR*(*F* ^2^) = 0.097
*S* = 1.02
6479 reflections
265 parameters
2 restraints
H-atom parameters constrained
Δρ_max_ = 1.01 e Å^−3^
Δρ_min_ = −1.38 e Å^−3^
Absolute structure: Flack (1983 ▶), 1997 Friedel pairs
Flack parameter: 0.024 (7)

### 

Data collection: *APEX2* (Bruker, 2005 ▶); cell refinement: *SAINT* (Bruker, 2005 ▶); data reduction: *SAINT*; program(s) used to solve structure: *SHELXTL* (Sheldrick, 2008 ▶); program(s) used to refine structure: *SHELXTL*; molecular graphics: *SHELXTL*; software used to prepare material for publication: *SHELXTL* and *PLATON* (Spek, 2003 ▶).

## Supplementary Material

Crystal structure: contains datablocks global, I. DOI: 10.1107/S1600536808043547/pk2136sup1.cif

Structure factors: contains datablocks I. DOI: 10.1107/S1600536808043547/pk2136Isup2.hkl

Additional supplementary materials:  crystallographic information; 3D view; checkCIF report

## Figures and Tables

**Table 1:** Hydrogen-bond geometry (Å, °)

*D*—H⋯*A*	*D*—H	H⋯*A*	*D*⋯*A*	*D*—H⋯*A*
C2—H2*A*⋯O1^i^	0.93	2.44	3.366 (4)	175
C4—H4*A*⋯O2^ii^	0.93	2.45	3.375 (4)	175
C6—H6*A*⋯O3^iii^	0.93	2.44	3.175 (4)	136
C7—H7*A*⋯O2^ii^	0.93	2.36	3.285 (4)	173
C14—H14*C*⋯O3^i^	0.96	2.36	3.304 (4)	168
C15—H15*A*⋯O2	0.96	2.57	3.515 (4)	168
C19—H19*A*⋯O1^iv^	0.93	2.47	3.306 (4)	149
C3—H3*A*⋯*Cg*3^ii^	0.93	2.76	3.601 (4)	151
C14—H14*B*⋯*Cg*2^v^	0.96	2.55	3.468 (4)	161

Symmetry codes: (i) 
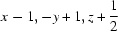
; (ii) 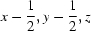; (iii) 
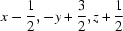
; (iv) 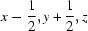; (v) 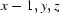. *Cg*2 and *Cg*3 are the centroids of the C8–C13 and C17–C22 rings.

**Fig. 1.**
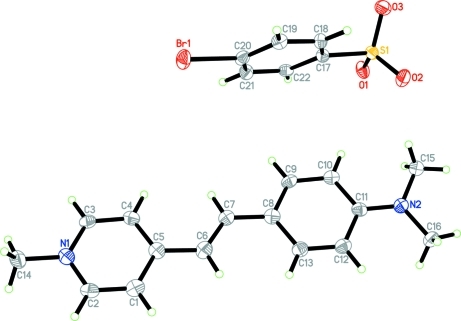
The asymmetric unit showing 50% probability displacement ellipsoids and the atom-numbering scheme.

**Fig. 2.**
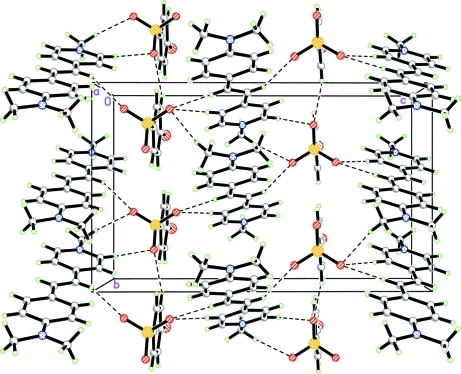
Crystal packing viewed along the a axis. The weak C—H···O interactions are drawn as dashed lines.

## supplementary crystallographic information

### Comment

Organic crystals with extensive conjugated π systems are attractive candidates for non-linear optic (NLO) studies because of their large NLO coefficients (Chia *et al.*, 1995; Dittrich *et al.*, 2003; Otero *et al.*, 2002; Nogi *et al.*, 2000; Sato *et al.*, 1999). 4-*N*,*N*-dimethylamino-4'-*N*'-methyl-stilbazolium tosylate (DAST) is one such promising NLO material (Adachi *et al.*, 1999). Previous studies (Dittrich *et al.*, 2003; Nogi *et al.*, 2000; Sato *et al.*, 1999) have shown that DAST and its analogues exhibit second-order non-linear optical properties. To investigate the effect of counter anion on the NLO properties of DAST, the title compound was prepared by changing the counter anion from 4-toluenesulfonate in DAST to 4-bromobenzenesulfonate. The title compound is found to crystallize with the non-centrosymmetric space group *C*c and therefore has second order nonlinear optical properties.

The asymmetric unit of the title compound (Fig. 1), consists of the C_16_H_19_N_2_^+^ cation and C_6_H_4_BrO_3_S^-^ anion. The cation exists in the *E* configuration with respect to the C6=C7 double bond [1.342 (5) Å] and the cation is nearly planar as indicated by the dihedral angle between the pyridinium and the dimethylaminophenyl rings 3.19 (15)°, and by the torsion angles C4–C5–C6–C7 = 1.0 (5)° and C6–C7–C8–C13 = 2.4 (5)°. Both methyl groups of the dimethylamino group are co-planar with the C8–C13 ring with the torsion angle C15—N2–C11–C10 of 2.3 (5)° and C16–N2–C11–C12 = 1.5 (5)°. The relative arrangement of cation and anion is shown by the angles between the mean plane of the 4-bromophenyl ring and those of the pyridinium and dimethylaminophenyl systems which are 74.81 (16)° and 77.99 (16)°, respectively. The bond lengths and angles are normal (Allen *et al.*, 1987) and the cation bond lengths and angles are comparable with related structures (Chantrapromma *et al.*, 2008).

In the crystal packing, all O atoms of sulfonate group are involved in weak C—H···O interactions (Table 1). The cations and anions are linked by weak C—H···O interactions into chains along the *c* axis and these chains are stacked along the *b* axis (Fig. 2). The crystal structure is further stabilized by a C—H···π interactions (Table 1). π–π interactions with the distance *Cg*_1_···*Cg*_2_ = 3.5675 (19) Å (symmetry code -1/2 + *x*, -1/2 + *y*, *z* and 1/2 + *x*, 1/2 + *y*, *z*) are observed; *Cg*_1_, *Cg*_2_ and *Cg*_3_ are the centroids of the N1/C1–C5, C8–C13 and C17–C22 rings.

### Experimental

(*E*)-4-[4-(Dimethylamino)styryl]-1-methylpyridinium iodide (compound A) was synthesized by mixing a solution (1:1:1 molar ratio) of 1,4-dimethylpyridinium iodide (2.00 g, 8.5 mmol), 4-dimethylaminobenzaldehyde (1.27 g, 8.5 mmol) and piperidine (0.84 ml, 8.5 mmol) in hot methanol (50 ml). The resulting solution was refluxed for 3 h under a nitrogen atmosphere. The resultant solid was filtered off, washed with diethylether to give red solid of compound A (2.86 g, 92%), Mp. 536–537 K). Silver(I) *p*-bromobenzenesulfonate (compound B) was synthesized according to our previously reported procedure (Chantrapromma *et al.*, 2006). The title compound was synthesized by mixing compound A (0.20 g, 0.5 mmol) in hot methanol (25 ml) and a solution of compound B (0.17 g, 0.5 mmol) in hot methanol (50 ml). The mixture immediately yielded a grey precipitate of silver iodide. After stirring the mixture for 30 min, the precipitate of silver iodide was removed and the resulting solution was evaporated yielding a red solid. Red plate-shaped single crystals of the title compound suitable for *x*-ray structure determination were recrystalized from methanol by slow evaporation of the solvent at room temperature over several days, Mp. 536–537 K.

### Refinement

All H atoms were placed in calculated positions with d(C—H) = 0.93 Å, *U*_iso_=1.2*U*_eq_(C) for aromatic and CH, 0.96 Å, *U*_iso_ = 1.5*U*_eq_(C) for CH_3_ atoms. A rotating group model was used for the methyl groups. The highest residual electron density peak is located at 0.47 Å from Br1 and the deepest hole is located at 0.71 Å from Br1. A total of 1997 Friedel pairs were used to determine the absolute structure.

### Crystal data

**Table:**

C_16_H_19_N_2_^+^·C_6_H_4_BrO_3_S^−^	*F*(000) = 976
*M**_r_* = 475.39	*D*_x_ = 1.548 Mg m^−^^3^
Monoclinic, *C**c*	Melting point = 536–537 K
Hall symbol: C -2yc	Mo *K*α radiation, λ = 0.71073 Å
*a* = 10.3712 (4) Å	Cell parameters from 6479 reflections
*b* = 10.9937 (5) Å	θ = 2.7–35.0°
*c* = 17.9027 (8) Å	µ = 2.15 mm^−^^1^
β = 92.442 (3)°	*T* = 100 K
*V* = 2039.37 (15) Å^3^	Plate, red
*Z* = 4	0.49 × 0.31 × 0.11 mm

### Data collection

**Table:**

Bruker SMART APEX2 CCD area-detector diffractometer	6479 independent reflections
Radiation source: fine-focus sealed tube	4811 reflections with *I* > 2σ(*I*)
graphite	*R*_int_ = 0.043
Detector resolution: 8.33 pixels mm^-1^	θ_max_ = 35.0°, θ_min_ = 2.7°
ω scans	*h* = −16→16
Absorption correction: multi-scan (*SADABS*; Bruker, 2005)	*k* = −17→14
*T*_min_ = 0.414, *T*_max_ = 0.787	*l* = −28→19
13146 measured reflections	

### Refinement

**Table:**

Refinement on *F*^2^	Secondary atom site location: difference Fourier map
Least-squares matrix: full	Hydrogen site location: inferred from neighbouring sites
*R*[*F*^2^ > 2σ(*F*^2^)] = 0.046	H-atom parameters constrained
*wR*(*F*^2^) = 0.097	*w* = 1/[σ^2^(*F*_o_^2^) + (0.022*P*)^2^ + 0.6614*P*] where *P* = (*F*_o_^2^ + 2*F*_c_^2^)/3
*S* = 1.02	(Δ/σ)_max_ = 0.001
6479 reflections	Δρ_max_ = 1.01 e Å^−^^3^
265 parameters	Δρ_min_ = −1.38 e Å^−^^3^
2 restraints	Absolute structure: Flack (1983), 1997 Friedel pairs
Primary atom site location: structure-invariant direct methods	Flack parameter: 0.024 (7)

### Special details

**Table:**

Experimental. The low-temperature data was collected with the Oxford Cryosystem Cobra low-temperature attachment.
Geometry. All e.s.d.'s (except the e.s.d. in the dihedral angle between two l.s. planes) are estimated using the full covariance matrix. The cell e.s.d.'s are taken into account individually in the estimation of e.s.d.'s in distances, angles and torsion angles; correlations between e.s.d.'s in cell parameters are only used when they are defined by crystal symmetry. An approximate (isotropic) treatment of cell e.s.d.'s is used for estimating e.s.d.'s involving l.s. planes.
Refinement. Refinement of *F*^2^ against ALL reflections. The weighted *R*-factor *wR* and goodness of fit *S* are based on *F*^2^, conventional *R*-factors *R* are based on *F*, with *F* set to zero for negative *F*^2^. The threshold expression of *F*^2^ > σ(*F*^2^) is used only for calculating *R*-factors(gt) *etc*. and is not relevant to the choice of reflections for refinement. *R*-factors based on *F*^2^ are statistically about twice as large as those based on *F*, and *R*- factors based on ALL data will be even larger.

### Fractional atomic coordinates and isotropic or equivalent isotropic displacement parameters (Å^2^)

**Table:**

	*x*	*y*	*z*	*U*_iso_*/*U*_eq_	
Br1	0.36598 (3)	0.77042 (3)	0.68824 (2)	0.02730 (9)	
S1	0.98401 (6)	0.80532 (7)	0.66253 (5)	0.01869 (16)	
O1	1.0332 (2)	0.6820 (2)	0.65917 (15)	0.0260 (5)	
O2	1.0299 (2)	0.8717 (2)	0.72875 (14)	0.0264 (5)	
O3	0.9990 (2)	0.8740 (2)	0.59428 (14)	0.0275 (5)	
N1	0.1505 (2)	0.3126 (2)	0.93738 (17)	0.0235 (6)	
N2	1.1241 (3)	0.6429 (3)	0.91479 (17)	0.0288 (6)	
C1	0.3252 (3)	0.3957 (3)	1.0096 (2)	0.0267 (7)	
H1A	0.3595	0.4215	1.0557	0.032*	
C2	0.2023 (3)	0.3507 (3)	1.0041 (2)	0.0248 (7)	
H2A	0.1538	0.3462	1.0466	0.030*	
C3	0.2213 (3)	0.3179 (3)	0.8760 (2)	0.0230 (7)	
H3A	0.1854	0.2908	0.8305	0.028*	
C4	0.3442 (3)	0.3621 (3)	0.8789 (2)	0.0238 (7)	
H4A	0.3909	0.3646	0.8357	0.029*	
C5	0.4003 (3)	0.4034 (3)	0.9463 (2)	0.0231 (7)	
C6	0.5317 (3)	0.4507 (3)	0.9554 (2)	0.0265 (8)	
H6A	0.5609	0.4749	1.0029	0.032*	
C7	0.6126 (3)	0.4612 (3)	0.8992 (2)	0.0231 (7)	
H7A	0.5821	0.4373	0.8519	0.028*	
C8	0.7443 (3)	0.5069 (3)	0.9063 (2)	0.0238 (7)	
C9	0.8152 (3)	0.5165 (3)	0.84225 (19)	0.0224 (7)	
H9A	0.7775	0.4917	0.7967	0.027*	
C10	0.9406 (3)	0.5618 (3)	0.8439 (2)	0.0229 (7)	
H10A	0.9842	0.5690	0.7998	0.027*	
C11	1.0015 (3)	0.5969 (3)	0.9124 (2)	0.0225 (7)	
C12	0.9307 (3)	0.5851 (3)	0.9777 (2)	0.0260 (7)	
H12A	0.9690	0.6066	1.0237	0.031*	
C13	0.8055 (3)	0.5421 (3)	0.9742 (2)	0.0250 (7)	
H13A	0.7608	0.5363	1.0179	0.030*	
C14	0.0170 (3)	0.2686 (3)	0.9326 (2)	0.0298 (8)	
H14A	0.0039	0.2197	0.8885	0.045*	
H14B	−0.0409	0.3367	0.9302	0.045*	
H14C	0.0007	0.2206	0.9760	0.045*	
C15	1.1928 (3)	0.6611 (3)	0.8465 (2)	0.0295 (8)	
H15A	1.1404	0.7084	0.8119	0.044*	
H15B	1.2110	0.5835	0.8246	0.044*	
H15C	1.2722	0.7032	0.8579	0.044*	
C16	1.1872 (3)	0.6788 (3)	0.9852 (2)	0.0304 (8)	
H16A	1.1782	0.6151	1.0213	0.046*	
H16B	1.1480	0.7518	1.0030	0.046*	
H16C	1.2771	0.6932	0.9781	0.046*	
C17	0.8138 (3)	0.7927 (3)	0.6710 (2)	0.0176 (6)	
C18	0.7402 (3)	0.8978 (3)	0.6780 (2)	0.0192 (7)	
H18A	0.7809	0.9732	0.6797	0.023*	
C19	0.6080 (3)	0.8917 (3)	0.6823 (2)	0.0209 (7)	
H19A	0.5594	0.9624	0.6858	0.025*	
C20	0.5489 (3)	0.7790 (3)	0.6814 (2)	0.0207 (6)	
C21	0.6187 (3)	0.6722 (3)	0.6750 (2)	0.0220 (7)	
H21A	0.5774	0.5970	0.6743	0.026*	
C22	0.7524 (3)	0.6800 (3)	0.6696 (2)	0.0191 (7)	
H22A	0.8008	0.6094	0.6649	0.023*	

### Atomic displacement parameters (Å^2^)

**Table:**

	*U*^11^	*U*^22^	*U*^33^	*U*^12^	*U*^13^	*U*^23^
Br1	0.01676 (11)	0.03575 (17)	0.02948 (18)	−0.00106 (18)	0.00199 (10)	−0.0056 (2)
S1	0.0163 (3)	0.0187 (4)	0.0211 (4)	0.0010 (2)	0.0013 (3)	0.0020 (3)
O1	0.0167 (9)	0.0242 (12)	0.0375 (16)	−0.0007 (8)	0.0055 (10)	0.0016 (11)
O2	0.0198 (10)	0.0342 (13)	0.0251 (14)	−0.0022 (9)	−0.0010 (9)	−0.0054 (11)
O3	0.0247 (10)	0.0308 (13)	0.0270 (14)	−0.0007 (9)	0.0021 (9)	0.0068 (10)
N1	0.0221 (12)	0.0201 (13)	0.0287 (18)	0.0065 (10)	0.0035 (12)	0.0037 (12)
N2	0.0305 (13)	0.0347 (16)	0.0212 (16)	−0.0087 (12)	0.0001 (12)	−0.0013 (14)
C1	0.0304 (15)	0.0215 (16)	0.028 (2)	0.0070 (12)	−0.0003 (14)	−0.0023 (14)
C2	0.0302 (15)	0.0235 (16)	0.0210 (19)	0.0080 (12)	0.0049 (14)	0.0013 (14)
C3	0.0269 (14)	0.0220 (16)	0.0197 (19)	0.0052 (12)	−0.0011 (13)	0.0028 (13)
C4	0.0249 (14)	0.0245 (16)	0.0223 (19)	0.0063 (12)	0.0044 (12)	0.0034 (14)
C5	0.0235 (13)	0.0176 (14)	0.0285 (19)	0.0062 (11)	0.0033 (13)	0.0004 (14)
C6	0.0229 (14)	0.0271 (17)	0.030 (2)	0.0064 (12)	0.0001 (14)	−0.0039 (15)
C7	0.0247 (14)	0.0203 (15)	0.0240 (19)	0.0036 (11)	−0.0007 (13)	−0.0008 (13)
C8	0.0245 (14)	0.0215 (15)	0.025 (2)	0.0047 (11)	0.0022 (13)	−0.0003 (14)
C9	0.0281 (14)	0.0203 (15)	0.0185 (18)	0.0035 (11)	−0.0031 (13)	−0.0020 (13)
C10	0.0265 (14)	0.0208 (15)	0.0214 (19)	0.0036 (11)	0.0017 (13)	−0.0004 (13)
C11	0.0284 (14)	0.0164 (14)	0.0228 (19)	0.0018 (11)	0.0008 (13)	0.0023 (13)
C12	0.0308 (15)	0.0245 (17)	0.0224 (19)	0.0005 (12)	−0.0014 (14)	−0.0009 (14)
C13	0.0277 (15)	0.0258 (16)	0.0217 (19)	0.0003 (12)	0.0023 (13)	−0.0035 (14)
C14	0.0250 (14)	0.0274 (16)	0.037 (2)	0.0018 (13)	0.0038 (14)	0.0079 (16)
C15	0.0299 (15)	0.0315 (18)	0.027 (2)	−0.0053 (13)	−0.0018 (14)	0.0038 (16)
C16	0.0354 (17)	0.0309 (18)	0.024 (2)	−0.0066 (14)	−0.0055 (15)	−0.0007 (16)
C17	0.0174 (12)	0.0168 (14)	0.0186 (18)	0.0008 (10)	0.0004 (12)	−0.0001 (12)
C18	0.0182 (12)	0.0177 (16)	0.0215 (19)	−0.0021 (11)	0.0002 (12)	−0.0015 (13)
C19	0.0229 (14)	0.0202 (16)	0.0197 (18)	0.0029 (11)	0.0012 (12)	−0.0028 (13)
C20	0.0167 (12)	0.0293 (16)	0.0160 (17)	0.0013 (12)	0.0003 (11)	−0.0020 (14)
C21	0.0230 (14)	0.0181 (16)	0.025 (2)	−0.0009 (11)	−0.0009 (13)	−0.0004 (14)
C22	0.0226 (14)	0.0150 (15)	0.0197 (19)	0.0036 (11)	−0.0001 (13)	0.0039 (13)

### Geometric parameters (Å, °)

**Table:**

Br1—C20	1.909 (3)	C9—H9A	0.9300
S1—O3	1.450 (3)	C10—C11	1.409 (5)
S1—O1	1.451 (2)	C10—H10A	0.9300
S1—O2	1.455 (2)	C11—C12	1.413 (5)
S1—C17	1.784 (3)	C12—C13	1.380 (4)
N1—C3	1.349 (4)	C12—H12A	0.9300
N1—C2	1.355 (4)	C13—H13A	0.9300
N1—C14	1.466 (4)	C14—H14A	0.9600
N2—C11	1.367 (4)	C14—H14B	0.9600
N2—C16	1.451 (4)	C14—H14C	0.9600
N2—C15	1.455 (5)	C15—H15A	0.9600
C1—C2	1.367 (5)	C15—H15B	0.9600
C1—C5	1.405 (5)	C15—H15C	0.9600
C1—H1A	0.9300	C16—H16A	0.9600
C2—H2A	0.9300	C16—H16B	0.9600
C3—C4	1.363 (4)	C16—H16C	0.9600
C3—H3A	0.9300	C17—C18	1.392 (4)
C4—C5	1.393 (5)	C17—C22	1.393 (4)
C4—H4A	0.9300	C18—C19	1.378 (4)
C5—C6	1.461 (4)	C18—H18A	0.9300
C6—C7	1.342 (5)	C19—C20	1.382 (5)
C6—H6A	0.9300	C19—H19A	0.9300
C7—C8	1.455 (4)	C20—C21	1.387 (5)
C7—H7A	0.9300	C21—C22	1.396 (4)
C8—C9	1.393 (5)	C21—H21A	0.9300
C8—C13	1.402 (5)	C22—H22A	0.9300
C9—C10	1.391 (4)		
			
O3—S1—O1	113.63 (15)	C10—C11—C12	117.7 (3)
O3—S1—O2	112.50 (15)	C13—C12—C11	121.0 (3)
O1—S1—O2	113.53 (14)	C13—C12—H12A	119.5
O3—S1—C17	104.72 (14)	C11—C12—H12A	119.5
O1—S1—C17	106.37 (13)	C12—C13—C8	121.6 (3)
O2—S1—C17	105.09 (15)	C12—C13—H13A	119.2
C3—N1—C2	119.8 (3)	C8—C13—H13A	119.2
C3—N1—C14	120.8 (3)	N1—C14—H14A	109.5
C2—N1—C14	119.4 (3)	N1—C14—H14B	109.5
C11—N2—C16	120.8 (3)	H14A—C14—H14B	109.5
C11—N2—C15	120.8 (3)	N1—C14—H14C	109.5
C16—N2—C15	118.3 (3)	H14A—C14—H14C	109.5
C2—C1—C5	120.8 (3)	H14B—C14—H14C	109.5
C2—C1—H1A	119.6	N2—C15—H15A	109.5
C5—C1—H1A	119.6	N2—C15—H15B	109.5
N1—C2—C1	120.5 (3)	H15A—C15—H15B	109.5
N1—C2—H2A	119.7	N2—C15—H15C	109.5
C1—C2—H2A	119.7	H15A—C15—H15C	109.5
N1—C3—C4	121.6 (3)	H15B—C15—H15C	109.5
N1—C3—H3A	119.2	N2—C16—H16A	109.5
C4—C3—H3A	119.2	N2—C16—H16B	109.5
C3—C4—C5	120.3 (3)	H16A—C16—H16B	109.5
C3—C4—H4A	119.8	N2—C16—H16C	109.5
C5—C4—H4A	119.8	H16A—C16—H16C	109.5
C4—C5—C1	117.0 (3)	H16B—C16—H16C	109.5
C4—C5—C6	124.4 (3)	C18—C17—C22	119.2 (3)
C1—C5—C6	118.6 (3)	C18—C17—S1	119.4 (2)
C7—C6—C5	123.9 (3)	C22—C17—S1	121.4 (2)
C7—C6—H6A	118.1	C19—C18—C17	121.0 (3)
C5—C6—H6A	118.1	C19—C18—H18A	119.5
C6—C7—C8	125.4 (3)	C17—C18—H18A	119.5
C6—C7—H7A	117.3	C18—C19—C20	119.0 (3)
C8—C7—H7A	117.3	C18—C19—H19A	120.5
C9—C8—C13	117.2 (3)	C20—C19—H19A	120.5
C9—C8—C7	118.8 (3)	C19—C20—C21	121.8 (3)
C13—C8—C7	124.0 (3)	C19—C20—Br1	119.0 (2)
C10—C9—C8	122.4 (3)	C21—C20—Br1	119.1 (2)
C10—C9—H9A	118.8	C20—C21—C22	118.5 (3)
C8—C9—H9A	118.8	C20—C21—H21A	120.8
C9—C10—C11	120.0 (3)	C22—C21—H21A	120.8
C9—C10—H10A	120.0	C17—C22—C21	120.5 (3)
C11—C10—H10A	120.0	C17—C22—H22A	119.7
N2—C11—C10	120.7 (3)	C21—C22—H22A	119.7
N2—C11—C12	121.6 (3)		
			
C3—N1—C2—C1	0.7 (5)	C9—C10—C11—C12	−0.5 (4)
C14—N1—C2—C1	−177.9 (3)	N2—C11—C12—C13	177.9 (3)
C5—C1—C2—N1	0.0 (5)	C10—C11—C12—C13	−0.8 (5)
C2—N1—C3—C4	−0.7 (5)	C11—C12—C13—C8	0.9 (5)
C14—N1—C3—C4	177.9 (3)	C9—C8—C13—C12	0.3 (5)
N1—C3—C4—C5	0.0 (5)	C7—C8—C13—C12	−179.9 (3)
C3—C4—C5—C1	0.7 (4)	O3—S1—C17—C18	−61.5 (3)
C3—C4—C5—C6	179.1 (3)	O1—S1—C17—C18	177.9 (3)
C2—C1—C5—C4	−0.7 (5)	O2—S1—C17—C18	57.2 (3)
C2—C1—C5—C6	−179.2 (3)	O3—S1—C17—C22	117.4 (3)
C4—C5—C6—C7	1.0 (5)	O1—S1—C17—C22	−3.2 (3)
C1—C5—C6—C7	179.4 (3)	O2—S1—C17—C22	−123.8 (3)
C5—C6—C7—C8	−179.5 (3)	C22—C17—C18—C19	−1.0 (5)
C6—C7—C8—C9	−177.8 (3)	S1—C17—C18—C19	178.0 (3)
C6—C7—C8—C13	2.4 (5)	C17—C18—C19—C20	1.5 (5)
C13—C8—C9—C10	−1.7 (5)	C18—C19—C20—C21	−1.1 (5)
C7—C8—C9—C10	178.5 (3)	C18—C19—C20—Br1	179.5 (3)
C8—C9—C10—C11	1.8 (5)	C19—C20—C21—C22	0.2 (5)
C16—N2—C11—C10	−179.9 (3)	Br1—C20—C21—C22	179.6 (3)
C15—N2—C11—C10	2.3 (5)	C18—C17—C22—C21	0.1 (5)
C16—N2—C11—C12	1.5 (5)	S1—C17—C22—C21	−178.9 (3)
C15—N2—C11—C12	−176.4 (3)	C20—C21—C22—C17	0.4 (5)
C9—C10—C11—N2	−179.2 (3)		

### Hydrogen-bond geometry (Å, °)

**Table:**

*D*—H···*A*	*D*—H	H···*A*	*D*···*A*	*D*—H···*A*
C2—H2A···O1^i^	0.93	2.44	3.366 (4)	175
C4—H4A···O2^ii^	0.93	2.45	3.375 (4)	175
C6—H6A···O3^iii^	0.93	2.44	3.175 (4)	136
C7—H7A···O2^ii^	0.93	2.36	3.285 (4)	173
C14—H14C···O3^i^	0.96	2.36	3.304 (4)	168
C15—H15A···O2	0.96	2.57	3.515 (4)	168
C19—H19A···O1^iv^	0.93	2.47	3.306 (4)	149
C3—H3A···*Cg*3^ii^	0.93	2.76	3.601 (4)	151
C14—H14B···*Cg*2^v^	0.96	2.55	3.468 (4)	161
C16—H16A···*Cg*3^vi^	0.93	2.98	3.446 (4)	111

Symmetry codes: (i) *x*−1, −*y*+1, *z*+1/2; (ii) *x*−1/2, *y*−1/2, *z*; (iii) *x*−1/2, −*y*+3/2, *z*+1/2;  (iv) *x*−1/2, *y*+1/2, *z*; (v) *x*−1, *y*, *z*; (vi) *x*+1/2, −*y*+3/2, *z*+1/2.
